# Can a One-Item Mood Scale Do the Trick? Predicting Relapse over 5.5-Years in Recurrent Depression

**DOI:** 10.1371/journal.pone.0046796

**Published:** 2012-10-03

**Authors:** Gerard D. van Rijsbergen, Claudi L. H. Bockting, Matthias Berking, Maarten W. J. Koeter, Aart H. Schene

**Affiliations:** 1 Department of Clinical Psychology, University of Groningen, Groningen, The Netherlands; 2 Department of Clinical Psychology and Psychotherapy, University of Marburg, Marburg, Germany; 3 Department of Psychiatry, Academic Medical Center, University of Amsterdam, Amsterdam, The Netherlands; University of Adelaide, Australia

## Abstract

**Background:**

To examine whether a simple Visual Analogue Mood Scale (VAMS) is able to predict time to relapse over 5.5-years.

**Methodology/Principal Findings:**

187 remitted recurrently depressed out-patients were interviewed using the Structured Clinical Interview for DSM-IV Axis I Disorders (SCID-I) and the 17-item Hamilton Depression rating scale (HAM-D) to verify remission status (HAM-D <10). All patients rated their current mood with the help of a Visual Analogue Mood Scale (VAMS) at baseline and at a follow-up assessment three months later. Relapse over 5.5-years was assessed by the SCID-I. Cox regression revealed that both the VAMS at baseline and three months later significantly predicted time to relapse over 5.5-years. Baseline VAMS even predicted time to relapse when the number of previous depressive episodes and HAM-D scores were controlled for. The baseline VAMS explained 6.3% of variance in time to relapse, comparable to the HAM-D interview.

**Conclusions/Significance:**

Sad mood after remission appears to play a pivotal role in the course of depression. Since a simple VAMS predicted time to relapse, the VAMS might be an easy and time-effective way to monitor mood and risk of early relapse, and offers possibilities for daily monitoring using e-mail and SMS.

**Trial Registration:**

International Standard Randomized Controlled Trial Register Identifier: ISRCTN68246470.

## Introduction

Major Depressive Disorder (MDD) is a recurrent disorder with 80% risk of relapse in the absence of adequate treatment [1]. After remission, residual symptoms are a consistent predictor of relapse [2], [3]. Since residual symptoms are known to fluctuate within patients [4], thorough and frequent monitoring after remission is important in order to detect potential relapse.

The semi-structured Hamilton Depression rating scale (HAM-D) interview [5] is one of the most frequently used instruments to monitor patients and assess depressive symptomatology [6]. Higher scores on the HAM-D have been found to predict relapse [7]–[10]. Nevertheless, administration of the HAM-D has some disadvantages including its extensive length (similar to self-report measures; IDS-SR: 30 items, BDI-II: 21 items), time required for administration, and reliance on training [11], [12]. Unidimensional subscales (six up to 14 items) derived from the HAM-D were comparable to the total HAM-D in predicting which patients had remitted from acute MDD [13]–[18]. However, even less extensive versions of the HAM-D are time consuming and rely on training.

Depressed mood after remission appears to be an important symptom in the process of relapse. A recent study demonstrated that depressed mood after remission, as assessed by the Mood Spectrum Self-report Questionnaire, was predictive of relapse over 6 months in remitted patients [19]. Moreover, mood reactivity to a sad mood provocation (i.e. increases in sad mood) was a vulnerability factor in 48 remitted depressed patients [20], and predicted relapse over 5.5-years prospectively in 172 remitted recurrently depressed patients [21]. Depressed mood is also among the symptoms experienced in both the prodromal and the residual phase of depression [22]. This finding provides empirical support for the rollback theory in which residual symptoms are considered prodromal symptoms for the next depressive episode [22]–[24].

Mood can be assessed within one minute with a simple Visual Analogue Mood Scale (VAMS), on which patients rate their current mood by placing a single mark on a simple 10-centimeter line with ‘happy’ and ‘sad’ on either side. Indication of current mood using a 10-centimeter black line was found to correlate with HAM-D (*r* = .79; anchors ‘normal’ to ‘most depressed’) and BDI (*r* = .58; anchors ‘not at all depressed’ to ‘most depressed’) scores and was also able to detect clinical change [25]–[30]. Benefits of a VAMS include the limited amount of time and absence of clinical training required, easy comprehensibility, and easy application throughout daily life of a high risk patient. Moreover, the VAMS offers opportunities for mood monitoring via internet and text messages. We are unaware of any studies using a VAMS in the prediction of relapse in depression.

Therefore the current study is the first to examine a) whether a VAMS predicts time to relapse in depression over 5.5-years in patients currently in remission from MDD, and b) the amount of variance explained in predicting time to relapse by the VAMS alone as well as c) when added to the HAM-D interview and vice versa.

## Methods

### Participants

The current study was part of a Randomized Controlled Trial comparing the effectiveness of preventive Cognitive Therapy (CT) added to Treatment As Usual (TAU) and compared to TAU alone in the prevention of relapse. The protocol was approved by the Amsterdam Medical Center ethical board. All patients provided written informed consent prior to participation in the study (see [31], [32] for more details). All participants of the RCT participated in the current study. In order to participate in the study patients had a) experienced two or more Major Depressive Episodes (MDEs) in the previous five years and b) current remission of MDE for at least 10 weeks but no longer than two years both defined according to the *Diagnostic and Statistical Manual of Mental Disorders* (DSM–IV, [33]) and assessed with the Structured Clinical Interview for DSM–IV (SCID, [34]) administered by trained interviewers; and c) a current score of <10 on the HAM-D. Exclusion criteria were: current mania or hypomania or a history of bipolar illness, any psychotic disorder (current and previous), organic brain damage, alcohol or drug abuse, predominant anxiety disorder, recent electroconvulsion therapy, recent cognitive treatment or receiving CT at the start of the study, or current psychotherapy with a frequency of more than twice a month.

### Measures

#### Visual analogue mood scale

Patients were asked to rate their current mood at baseline and three months after baseline by placing a cross on a Visual Analogue Mood Scale (VAMS) with the following instruction: ‘You can answer the following question by placing a cross on the line from 0 to 10: at the moment I feel.’. The VAMS measured 100 mm between the two anchors with the descriptor “happy” located to the left of the center while “sad” was located on the right.

### 17-item Hamilton Depression Rating Scale

The 17-item Hamilton Depression rating scale (HAM-D, [5]) was used by telephone [35] to assess levels of depressive symptomatology at baseline and three months after baseline. This widely used semi-structured interview covers affective, behavioral and biological symptoms with scores that range between 0 and 52. The HAM-D was administered by trained research assistants and psychologists who were blind to treatment condition. Second ratings (*n* = 17) demonstrated high intraclass correlation (*r = *.94), indicating high agreement.

### Relapse/recurrence

The main outcome measure was time to relapse/recurrence assessed using the SCID-I. Current and past MDEs were checked for all patients at five assessment-points (3, 12, 24, 36 and 66 months). To keep the assessors blind with respect to treatment condition, we instructed participants not to reveal this information to the interviewers. Kappa (κ) for interrater agreement on relapse between the interviewers and an independent psychiatrist, assessed over the assessment period, ranged between 0.94 to 0.96, indicating excellent agreement.

### Use of Antidepressant Medication (ADM)

Patients were asked about their use of ADM for the last depression before entry of the study and whether they continued using ADM after remission [36], [37]. During the first two years of the study, every three months, information on ADM (type and dosage) over the previous month was monitored using the Trimbos/iMTA Self-Report Questionnaire for Costs associated with Psychiatric Illness [38], which covers a maximum recall period of one month. Additionally, information on continuous use was also collected by the interviewer during the 24, 36 and 66 month interviews retrospectively. Adherence was assessed with the Medication Adherence Questionnaire (MAQ) [39].

### Statistical Analysis

First of all, we were interested in the prediction of time to relapse within 5.5-years by the VAMS at the two available assessment-points (baseline and three months after baseline). We used survival analysis (Cox regression) in which patients who dropped-out or who did not relapse within 5.5-years were treated as censored. Since use of ADM and number of previous episodes might influence VAMS scores, we checked all models for confounding by these variables.

To assess whether CT moderated the relation between the predictors and relapse, we examined the two-way interaction of Condition x predictor and the three-way interaction of Condition x predictor x Previous MDEs since in a previous study the number of previous MDEs was a moderator of predictors of interest on relapse [31], [32]. If CT affected this relation the analyses was restricted to the control group only, otherwise the analysis was performed on the complete sample.

Moreover, we were interested in the amount of variance explained in time to relapse by the VAMS and the HAM-D interview. Since Cox regression does not provide a measure for explained variance directly, we calculated explained variance using Nagelkerke’s R^2^ [40] formula. We calculated explained variance for a model containing the VAMS only, as well as two combined models in which the HAM-D was entered in the first block whereas the VAMS was entered in the second block and vice versa.

## Results

### Patient Characteristics and Flow

In total 187 formerly depressed patients were included in the study. For the analyses we excluded 15 patients (drop-outs), resulting in a remaining 172 patients. Drop-outs were slightly younger (*t*(170) = −2.25, *p* = 0.03), but did not differ on any other characteristic.

Demographic and clinical characteristics are summarized in Table 1. Patients were predominantly female (74%) and were currently in remission of a highly recurrent MDD (median number of MDEs: 4.0) with 3.8 residual symptoms (HAM-D) on average.

**Table 1:** Demographic and Clinical Characteristics at Baseline.

	TAU (n = 84)	TAU + CT (n = 88)
Demographics^a^		
Female % (n)	74.0 (62)	73.0 (64)
Age	43.4 (9.8)	45.9 (9.1)
Median previous episodes (IQR)	3.0 (3.8)	4.0 (3.8)
Age of first onset	28.1 (12.5)	28.9 (12.6)
Patients on antidepressants% (n)	50.0 (42.0)	52.0 (46.0)
Clinical characteristics		
VAMS_baseline_	37.0 (21.0)	34.0 (20.0)
VAMS_three months_	33.0 (19.0)	32.0 (18.0)
Total HAM-D_17_	3.7 (2.9)	3.8 (2.8)

*Note.* TAU = Treatment As Usual, CT = Cognitive Therapy, IQR = Interquartile Range, VAMS = Visual Analogue Mood Scale.

a All values represent mean (SD) unless stated otherwise.

### Preliminary VAMS Analyses

The VAMS at baseline and three months later demonstrated moderate stability and were significantly associated (*r* = .30; *p*<0.001). After controlling for depressive symptoms (HAM-D) at three months after baseline, both VAMS measurements remained significantly associated (*r* = .25; *p* = 0.001).

### Prediction of Relapse Using the VAMS

Since none of the interaction terms were significant and neither the number of previous MDEs nor use of ADM confounded the predictor of interest, interaction terms were subsequently dropped from the model and Cox regression was fitted with the individual predictor only (see Table S1, S2 for interaction coefficients).

Cox regression revealed that the baseline VAMS significantly predicted time to relapse in 5.5-years (Wald χ (2,1) = 11.758, *p = *0.001, hazard ratio = 1.15). Each centimeter increase on the VAMS increased the prospective risk of relapse with a factor 1.15 (15%). The baseline VAMS remained a significant predictor up and above the most consistent predictors of relapse, the total HAM-D and the number of previous MDEs.

In order to replicate prediction of relapse by a single VAMS in the current sample, we subsequently investigated whether a single VAMS administered three months after baseline was also predictive of relapse. Again, a single VAMS predicted time to relapse in 5.5-years (Wald χ (2,1) = 7.091, *p = *0.008, hazard ratio = 1.12), though this time not up and above the total HAM-D.

### Comparing Explained Variance of the Baseline VAMS to the HAM-D

A previous study on this sample already demonstrated that the total baseline HAM-D predicted time to relapse, and explained 6.0% in variance of time to relapse over 5.5-years [10].

We were now interested in the amount of variance explained by the baseline VAMS, which was 6.3% (comparable between conditions, 5.5% for TAU and 6.8% for TAU+CT). Second, when the VAMS was added to a model already containing the HAM-D, explained variance of the model increased from 6.0% to 10.3%. Likewise, when the total HAM-D was added to a model already containing the VAMS, an additional 4.1% was explained.

## Discussion

The current study focused on the question whether a simple Visual Analogue Mood Scale (VAMS) predicted time to relapse in depression over 5.5-years, and how well the VAMS predicted time to relapse compared to the HAM-D.

The current study demonstrated that even a simple VAMS was able to predict time to relapse for 5.5-years in recurrently depressed patients currently in remission. Both VAMS measurements (at baseline and three months later) predicted time to relapse and were significantly associated to each other. The baseline VAMS even predicted relapse up and above a frequently used depression interview, i.e. the HAM-D interview. Our findings stress the relevance of mood as a risk factor for relapse, which is in line with previous studies [19]–[21].

Moreover, the baseline VAMS predicted time to relapse for 5.5-years comparable to the HAM-D interview in terms of explained variance. When the VAMS was added to the HAM-D, additional variance was explained. However, the same was true when HAM-D was added to the VAMS, suggesting differences in what both instruments measure. Nevertheless, the predictive power of the VAMS as well as the HAM-D was indicative of a small effect size (6% in variance). Potentially, when assessed repeatedly, explained variance of the VAMS might increase.

Previous use of a Visual Analogue Scale in patients already demonstrated high convergent validity (*r* = .85) and test re-test reliability (*r* = .96) in the improvement of winter depression in 162 patients receiving light therapy [41]. Furthermore, a Visual Analogue Scale was also able to detect depressive symptoms among 157 elderly patients with cognitive impairments [42], but also in patients suffering from somatic illnesses including coronary syndrome [43] and diabetes mellitus [44]. The current study extends the use of a VAMS to the prediction of relapse in depression. The VAMS is easily administered in less than a minute and might be a feasible way to assess patients at high risk of relapse in clinical practice. Moreover, the VAMS could be used in combination with the HAM-D in stepwise monitoring, as a tool for early detection of relapse using new devices including mobile phone applications, short message service and e-mail monitoring [45]–[48]. When mood on the VAMS appears to be low, monitoring could be intensified using the HAM-D interview.

Several limitations of the current study have to be noted. First of all, since Cox regression does not provide explained variance directly, explained variance had to be calculated using Nagelkerke’s R^2^ [40]. The amount of variance explained should therefore be considered as an estimation. Second, although we checked our analyses for confounding by baseline use of ADM, we cannot completely rule out the potential influence of ADM on prediction by the VAMS later in the study since actual use was monitored only twice (retrospectively) in the final 3.5-years of the study. Third, the three-month stability of the VAMS used in the current study was maximal moderate. The use of this simple and quick screening instrument might thus result in decreases in reliability. Finally, lack of standardization of the VAMS has resulted in many different one-item mood scales. While in the current study, similar to previous studies [49]–[51], ‘happy’ and ‘sad’ were used as anchors, other anchors have been described in the literature as well, i.e. ‘neutral’ to ‘sad’, ‘not at all depressed’ to ‘most depressed’, ‘worst mood’ to ‘best mood’ [26]–[28], [52], which makes it difficult to compare the VAMS among studies and could explain differences in results. It is currently unknown which anchors are most reliable in measuring mood. Future studies should therefore focus on optimizing the VAMS scale by determining the most sensitive and reliable anchor points. Furthermore, since previous studies indicate that mood and fluctuations in mood are related to relapse [19]–[21] studies should include repeated assessment of mood (daily sampling) to study the stability of mood and thereby enhance early detection of relapse.

## Supporting Information

Table S1 **Cox Regression Model on Prediction of Relapse Including Three-way Interaction of Predictor x Condition x Previous MDEs (n = 172).**
(DOCX)

Table S2 **Cox Regression Model on Prediction of Relapse Including Two-way Interaction of Predictor x Condition (n = 172).**
(DOCX)
