# Supplementary material for: Can a One-Item Mood Scale Do the Trick? Predicting Relapse over 5.5-Years in Recurrent Depression
Source: PLoS One. 2012 Oct 3;7(10):e46796. doi: 10.1371/journal.pone.0046796 (PMC3463530; doi:10.1371/journal.pone.0046796)
Supplement: Table S1 — Cox Regression Model on Prediction of Relapse Including Three-way Interaction of Predictor x Condition x Previous MDEs (n = 172). (DOCX) [file pone.0046796.s001.docx]

**Supporting Information**

Table S1

|  | Predictor  (β_1_) | Condition  (β_2_) | Number of previous MDEs (β_3_) | Number of previous MDEs x predictor (β_4_) | Number of previous MDEs x condition  (β_5_) | Predictor x previous MDEs x condition  (β_6_) |
| --- | --- | --- | --- | --- | --- | --- |
| VAMS_baseline_  β  SE (β)  *p* | 0.124  0.067  0.065 | -0.219  0.314  0.485 | -0.045  0.206  0.826 | 0.014  0.055  0.802 | 0.579  0.313  0.064 | -0.022  0.059  0.715 |
| VAMS_three months_  β  SE (β)  *p* | 0.126  0.066  0.055 | -0.362  0.317  0.253 | -0.025  0.209  0.905 | -0.019  0.053  0.723 | 0.694  0.341  0.042 | -0.020  0.064  0.758 |

*Note.* VAMS = Visual Analogue Mood Scale
