# Supplementary material for: Can a One-Item Mood Scale Do the Trick? Predicting Relapse over 5.5-Years in Recurrent Depression
Source: PLoS One. 2012 Oct 3;7(10):e46796. doi: 10.1371/journal.pone.0046796 (PMC3463530; doi:10.1371/journal.pone.0046796)
Supplement: Table S2 — Cox Regression Model on Prediction of Relapse Including Two-way Interaction of Predictor x Condition (n = 172). (DOCX) [file pone.0046796.s002.docx]

**Supporting Information**

Table S2

|  | Predictor  (β_1_) | Condition  (β_2_) | Condition x predictor (β_3_) |
| --- | --- | --- | --- |
| VAMS_baseline_  β  SE (β)  *p* | 0.153  0.061  0.012 | 0.382  0.341  0.263 | -0.029  0.081  0.718 |
| VAMS_three months_  β  SE (β)  *p* | 0.121  0.065  0.062 | 0.355  0.331  0.283 | -0.021  0.084  0.799 |

*Note.* VAMS = Visual Analogue Mood Scale
